# Application of red light phototherapy in the treatment of radioactive dermatitis in patients with head and neck cancer

**DOI:** 10.1186/s12957-018-1522-3

**Published:** 2018-11-12

**Authors:** Xudong Zhang, Hongfei Li, Qian Li, Ying Li, Chao Li, Minmin Zhu, Bing Zhao, Guowen Li

**Affiliations:** Radiotherapy inpatient Ward II, The First Affiliated Hospital of Zhengzhou University, No.1 Eastern Jianshe Road, Zhengzhou, 450000 Henan China

**Keywords:** Radioactive dermatitis, Red light phototherapy, Nasopharyngeal carcinoma, Head and neck cancer

## Abstract

**Background:**

To observe the effect of red light phototherapy (RLPT) on radioactive dermatitis (RD) caused by radiotherapy in patients with head and neck cancer (HNC).

**Methods:**

Sixty patients with HNC admitted to our hospital were randomly divided into experimental group and control group, 30 patients in each group. The control group received routine daily care during radiotherapy treatment. In the experimental group, in addition to routine daily care during radiotherapy treatment, photon therapy apparatus RLPT was added, 10 min/time, 2 times/day, and lasted until the end of radiotherapy. The pain and conditions of the patients’ skin were assessed daily, and the skin pain and dermatitis grades of the two groups were compared.

**Results:**

In terms of the reaction degree of RD, experimental group was mainly grade 0–2, and control group was mainly grade 2–3, with a significant difference (*P* < 0.05). In terms of skin pain, according to the pain records at week 2, 3, and 4, the pain degree increased with time. However, the score of wound pain in experimental group was significantly lower than that in control group, and there was a significant difference between the two groups (*P* < 0.05).

**Conclusions:**

The application of RLPT in the treatment of RD can help accelerate wound healing and significantly shorten healing time. It can not only reduce wounds pain of patients, promote inflammation and ulcer healing, but also ensure the smooth progress of patients’ radiotherapy and improve their quality of lives, which is worth popularization and application in the clinical practice.

## Background

Head and neck cancer (HNC), represented by nasopharyngeal carcinoma (NPC), is one of the most frequent cancers in China and Southeast Asia countries, and its incidence is increasing gradually. Due to its anatomical and pathological characteristics, radiotherapy is still the main method to treat NPC [1]. However, radiation-induced skin reaction is the most common complication of tumor radiotherapy, and its incidence is high. About 87% of patients with radiotherapy will have erythema and more serious radioactive skin reactions [2, 3]. Radioactive dermatitis (RD) is mainly caused by skin exposure to high energy physical radiation, resulting in skin mucosal inflammatory damage. It manifests as erythema, epithelial shedding, skin ulcers, and pain. Severe cases can cause local or systemic infection. As the red light of visible light (the wavelength is 600–700 nm), photochemical effect has a physiotherapy effect on the body [4]. The application of red light phototherapy (RLPT) to systemic burn wounds has achieved good results in relieving pain and preventing cross infection [5, 6]. Based on the clinical practice of our hospital, we have done some summative research to confirm the positive therapeutic effect of RLPT on RD.

## Methods

### Patients

**Fig. 1:**
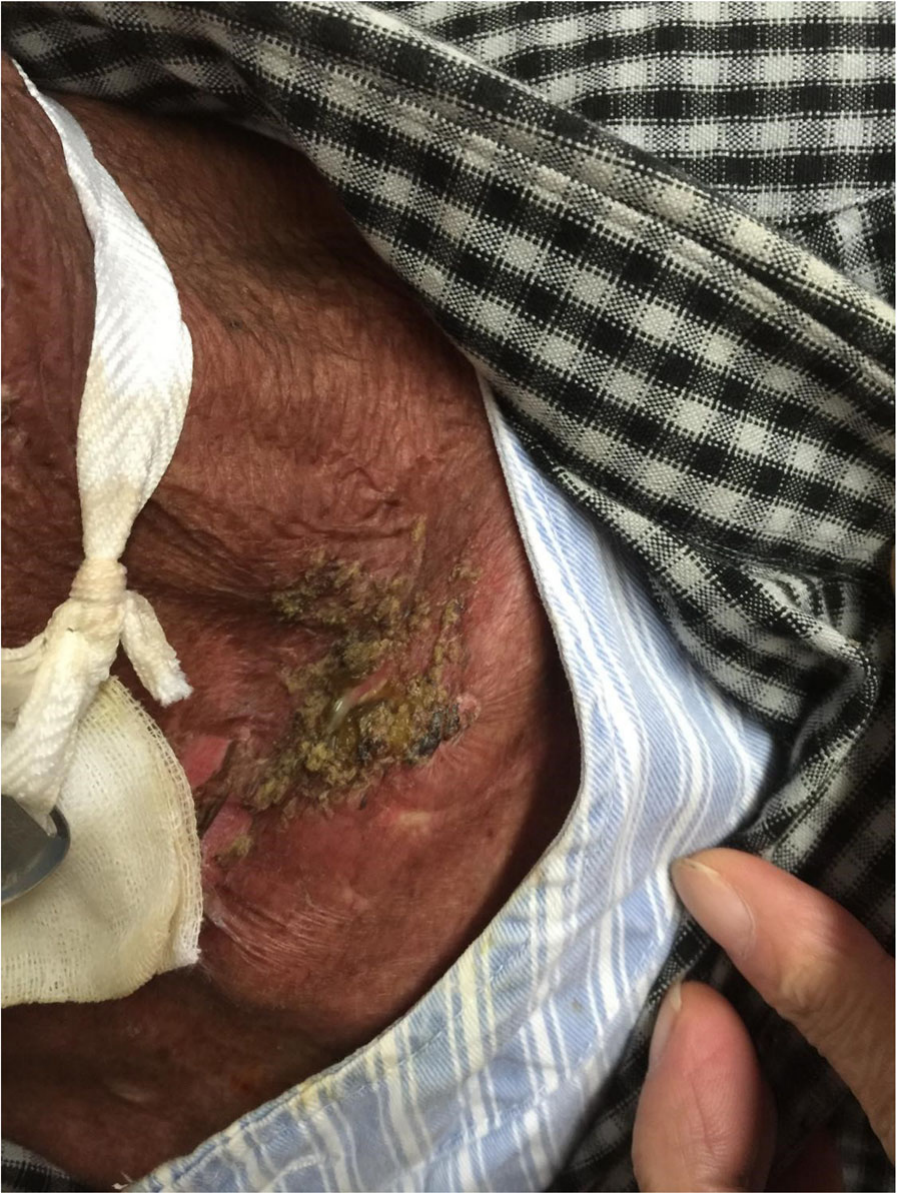
Patient with severe radioactive dermatitis, the patient stated that skin was painful, and skin surface ulceration and secretion could be seen. Before irradiation

Sixty patients with HNC admitted to our hospital from January 2017 to July 2017 were selected in this research. Among them, 52 cases were NPC, 4 cases were laryngeal cancer, 2 cases were tonsillar carcinoma, and 2 cases were tongue cancer. And males were 42 cases, accounting for 70%; females were 18 cases, accounting for 30%; aged 24–75 years. In addition, education background below junior high school was 14 cases, accounting for 23%; education background above junior high school was 46 cases, accounting for 77%. All patients received three-dimensional intensity-modulated radiation therapy with 30 to 32 irradiations for 6 weeks. The study not only received informed consent from all patients, but also received the support of the ethics committee of the First Affiliated Hospital of Zhengzhou University (Fig. 1).

Inclusion criteria were ① pathologically diagnosed; ② received chemotherapy and radiotherapy for the first time; and ③ signed the informed consent and willing to involve in this research.

Exclusion criteria were ① patients with communication disorders; and ② patients who were unwilling to take part in this treatment.

All patients were randomly divided into two groups, control group (*n* = 30) and experimental group (*n* = 30). There was no significant difference in the general data between the two groups, gender distribution (*P* > 0.05), educational background distribution (*P* > 0.05), and payment methods (*P* > 0.05). In terms of age distribution, the experimental group was 46.4 ± 11.91 years old, and the control group was 45.23 ± 12.70 years old; there was no significant difference between the two groups (*P* > 0.05) (Table 1).

**Table 1:** Comparison of general data between two groups

Item	Experimental group (*n* = 30)	Control group (*n* = 30)	*χ*^*2*^/*t*	*P*
Gender [*n* (%)]				
Male	22 (73.3)	20 (66.7)	0.317	0.574
Female	8 (26.7)	10 (33.3)		
Education background [n (%)]				
Below junior high school	6 (20)	8 (26.7)	0.373	0.523
Above junior high school	24 (80)	22 (73.3)		
Payment method [*n* (%)]				
Medical insurance	29 (96.7)	28 (93.3)		0.500^a^
Self-supporting	1 (3.3)	2 (6.7)		
Age	46.4 ± 11.91	45.23 ± 12.70	0.453	0.667

^a^Fisher’s exact test result, no chi-square value

### Methods

Control group: routine methods of nursing were given during radiotherapy, including health education, skin self-care, and skin protective agent. 0.9% normal saline cotton balls were used to gently clean the wound and remove necrotic tissue, and the wound were dried with sterile gauze.

Experimental group: in addition to gently cleaning the wound with 0.9% normal saline cotton ball to remove the necrotic tissue, the RLPT treatment was also used. The patient was in a supine position, and the radiation field skin was fully exposed. The irradiation time was 10 min, 2 times/day, the lampshade was 15–20 cm from the wound surface, and the wound temperature was 30 °C. In the process of irradiation, doctors and patients should wear sunglasses to avoid eye injuries caused by strong light and the doctors should ask the patients if they are uncomfortable in time. If there is any abnormality, they should timely handle it and record it.

### Observation index and curative effect evaluation

The degree of skin reaction and pain in the neck were observed daily during the treatment. Radioactive skin lesions were graded according to the acute radiation response scoring criterion of American Radiation Therapy Oncology Group [7]. Grade 0: no change; grade 1: follicular dark red spots, dry desquamation, depilation, hair loss, and sweat reduction; grade 2: tender or bright red spots, patchy erosion, and moderate edema; grade 3: external position erosion of skin wrinkles and pitting edema; grade 4: ulcer, bleeding, and necrosis. The degree of pain was assessed by the numerical rating scale (NRS), and the degree of pain was expressed as a number from 0 to 10. 0 was painless; 1–3 was mild pain, which can be tolerated; 4–6 was moderate pain, which is severely disturbed, accompanied by irritability or passive position [8]. The participants chose one of the numbers according to their personal pain feelings. NRS had good reliability and validity, and was easy to record.

### Statistical analysis

SPSS17.0 software was used for statistical analysis, and descriptive statistical analysis was performed on general data. Measurement data were expressed as mean ± standard deviation, and the difference between groups was tested by independent sample *t* test. Chi-square test was used to compare the difference between groups of enumeration data, and non-parametric rank sum test was used to compare the rank data. There was a significant difference at *P* < 0.05.

## Results

### Comparison of the degree of RD reaction between the two groups

In the experimental group and control group, there was a significant difference in the degree of RD reaction between the two groups. The experimental group was mainly composed of grade 0–2 RD, including 18 cases (60.00%) of grade 0–1 and 12 cases (40.00%) of grade 2. The control group was mainly composed of grade 2–3 RD, including 19 cases of grade 2 (63.33%), 9 cases of grade 3 (30.00%) and only 2 cases of grade 0–1 (6.67%). It showed that the degree of inflammatory response of the experimental group was lighter than that of the control group. There was a significant difference between the two groups (Table 2).

**Table 2:** Comparison of the degree of radioactive dermatitis reaction between the two groups (*n*)

Group	Number	Grade 0–1	Grade 2	Grade 3	*U*	*P*
Experimental group	30	18	12	0	4.79	0.000
Control group	30	2	19	9		

### Comparison of occurrence of skin pain at different times between the two groups

**Fig. 2:**
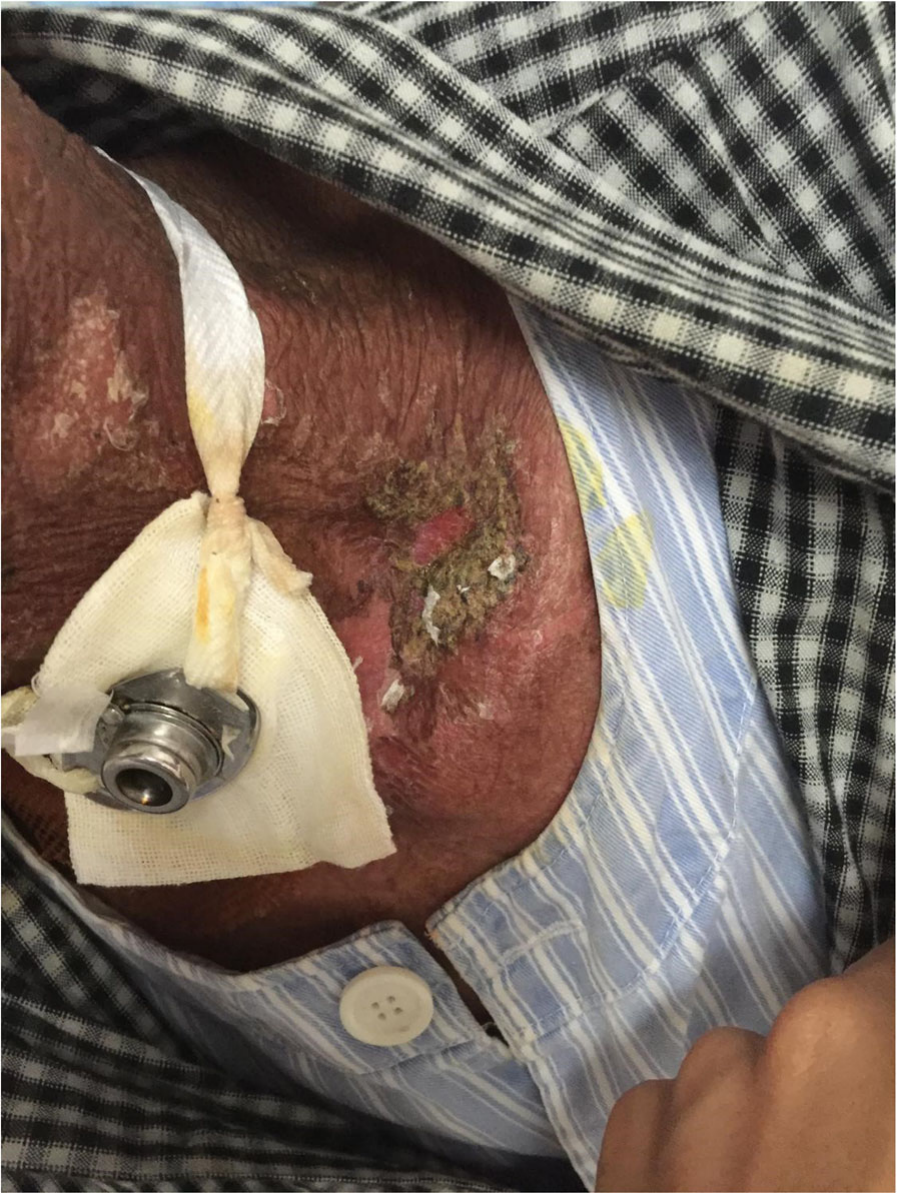
Patient with severe radioactive dermatitis, the patient stated that skin was painful, and skin surface ulceration and secretion could be seen. The second irradiation, the wound was basically dry and the pain was less than before

**Fig. 3:**
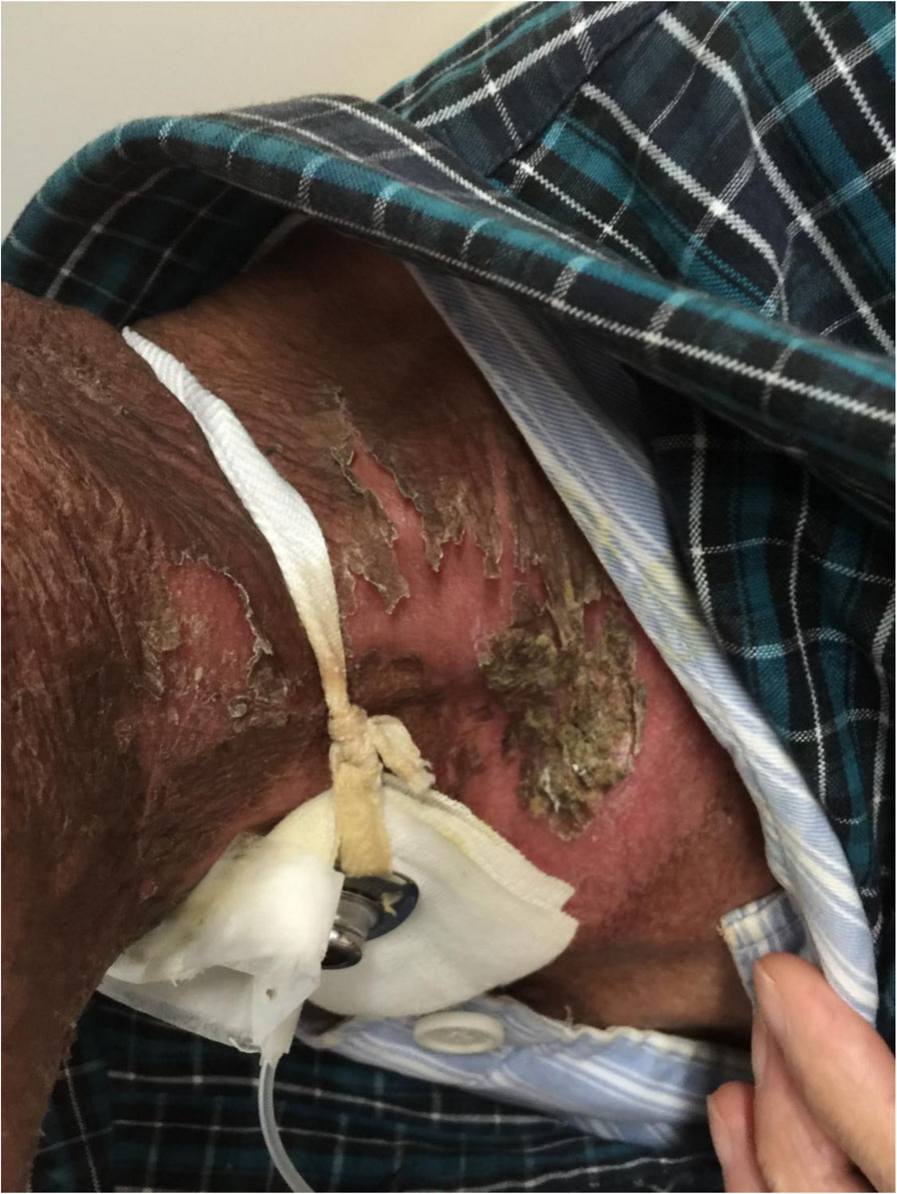
Patient with severe radioactive dermatitis, the patient stated that skin was painful, and skin surface ulceration and secretion could be seen. The fifth irradiation, the scabs came off, and pain was almost gone

**Fig. 4:**
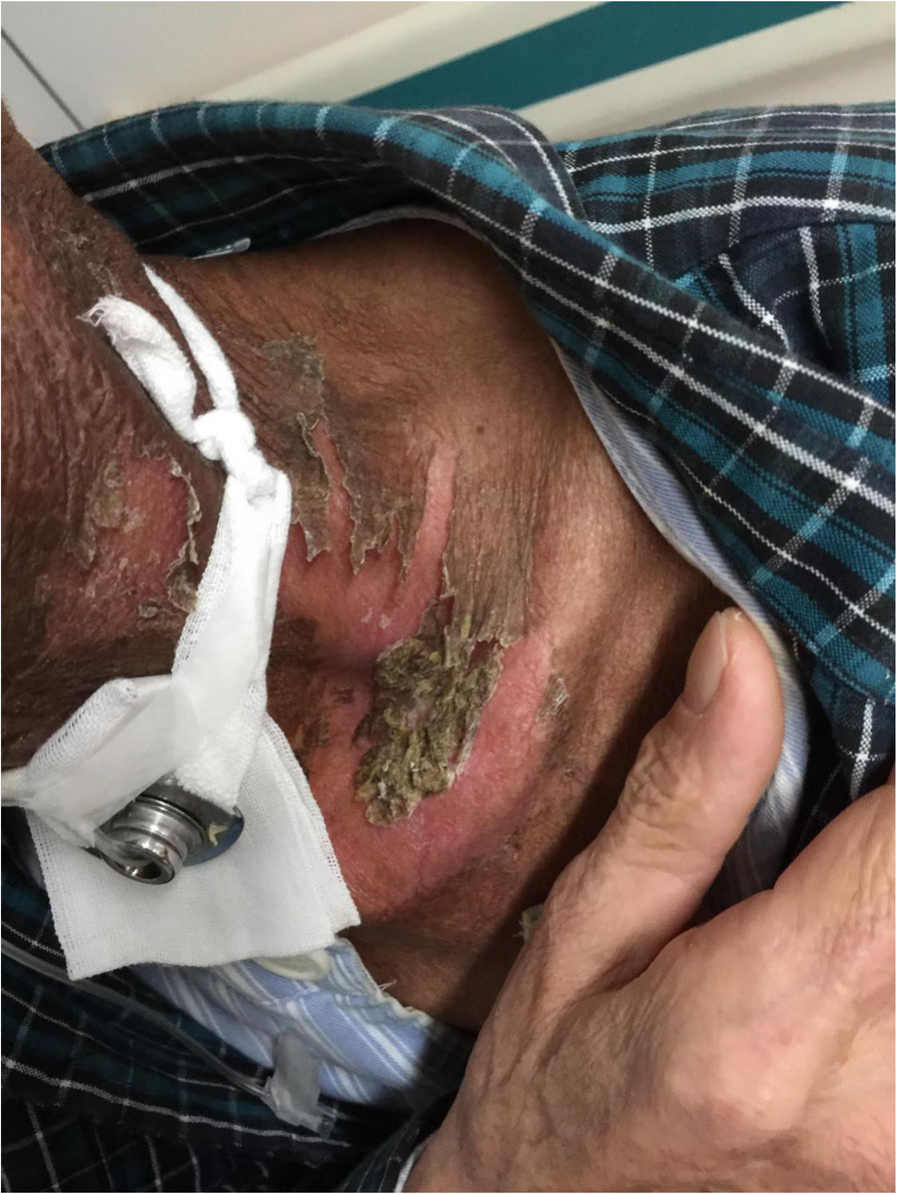
Patient with severe radioactive dermatitis, the patient stated that skin was painful, and skin surface ulceration and secretion could be seen. The sixth irradiation

In Table 3, the results showed that there was a significant difference in the occurrence of skin pain at the end of second, third, and fourth week between the two groups (*P* < 0.05), but there was no significant difference at the end of fifth and sixth week between the two groups (*P* > 0.05). Comparison within groups, the chi-square test results of occurrence of skin pain at different time points in the experimental group and the control group were *χ*^*2*^ = 65.083 and *χ*^*2*^ = 27.091, respectively, with significant differences (*P* < 0.05). It showed that there was a linear trend in each group, and the occurrence of skin pain increased with time (Figs. 2, 3, and 4).

**Table 3:** Comparison of occurrence of skin pain at different times between the two groups

	Group E (*n* = 30)	Group C (*n* = 30)	*χ* ^*2*^	*P*
End of second week				
Mild	2	16	15.56	0.000
Moderate	0	0		
Severe	0	0		
End of third week				
Mild	7	26	24.31	0.000
Moderate	0	0		
Severe	0	0		
End of fourth week				
Mild	16	26	7.94	0.005
Moderate	0	0		
Severe	0	0		
End of fifth week				
Mild	27	30	3.61	0.076
Moderate	0	0		
Severe	0	0		
End of sixth week				
Mild	27	27	1.65	0.098
Moderate	0	3		
Severe	0	0		
*χ* ^*2*^	65.083	27.091		
*P*	0.000	0.000		

Note: Group E is experimental group, and group C is control group

## Discussion

The incidence of HNC ranks sixth in all tumor types and mortality ranks eighth. Radiation therapy is still the main method for treating such cancers [9–11]. The skin reaction caused by radiation is the most common complication of tumor radiotherapy, and its incidence is high. About 87% of patients with radiotherapy will have erythema and more serious radiation skin reactions. For early lesions, 10-year disease-related survival rate, recurrence-free survival rate, and distant metastasis-free survival rate were 98%, 94%, and 98%, respectively. However, due to the high dose of radiotherapy, radiation would cause certain damage to the skin of the irradiated field to form RD [12–15].

RD is mainly due to the skin receives high energy physical radiation, which directly damage the human epidermal cell DNA molecules. It is an inflammatory damage of the skin mucosa caused by radiation (mainly β, γ, and *x* rays). It is characterized by erythema, epithelial shedding, skin ulcers, and pain. Severe cases can cause local or systemic infection. Acute radioactive skin reactions often cause itching and pain, and delays in treatment can affect appearance and lower quality of life [15–18].

Infrared therapy apparatus adopts high-energy semiconductor chip to integrate cold light source. Its specific wavelength red light photons and high efficient biochemical enzymatic reaction mechanism significantly stimulate fibroblast and endothelial cell growth, promote granulation formation, relieve pain, and accelerate wound healing. Specifically, long-wave infrared light can reach the shallow layer of the skin, while short-wave infrared light may reach deep skin or even subcutaneous tissue, and the red light band (620–760 am) can cause deep tissue vasodilation and circulation improvement [19]. After the application of close-range RLPT in RD, red light is strongly absorbed by the mitochondria of human cells. Through photochemical action, it promotes material metabolism, strengthens the cell activity, promotes the proliferation of epithelial tissue in the wound of the patient, improves the local blood circulation, and speeds up the formation of granulation tissue. On the basis of ensuring skin integrity, it promotes the healing of tissues, shortens the time of treatment, and relieves the pain of patients [20, 21]. In terms of safety, studies have shown that RLPT has little adverse reaction and even no adverse reactions occur [22].

**Fig. 5:**
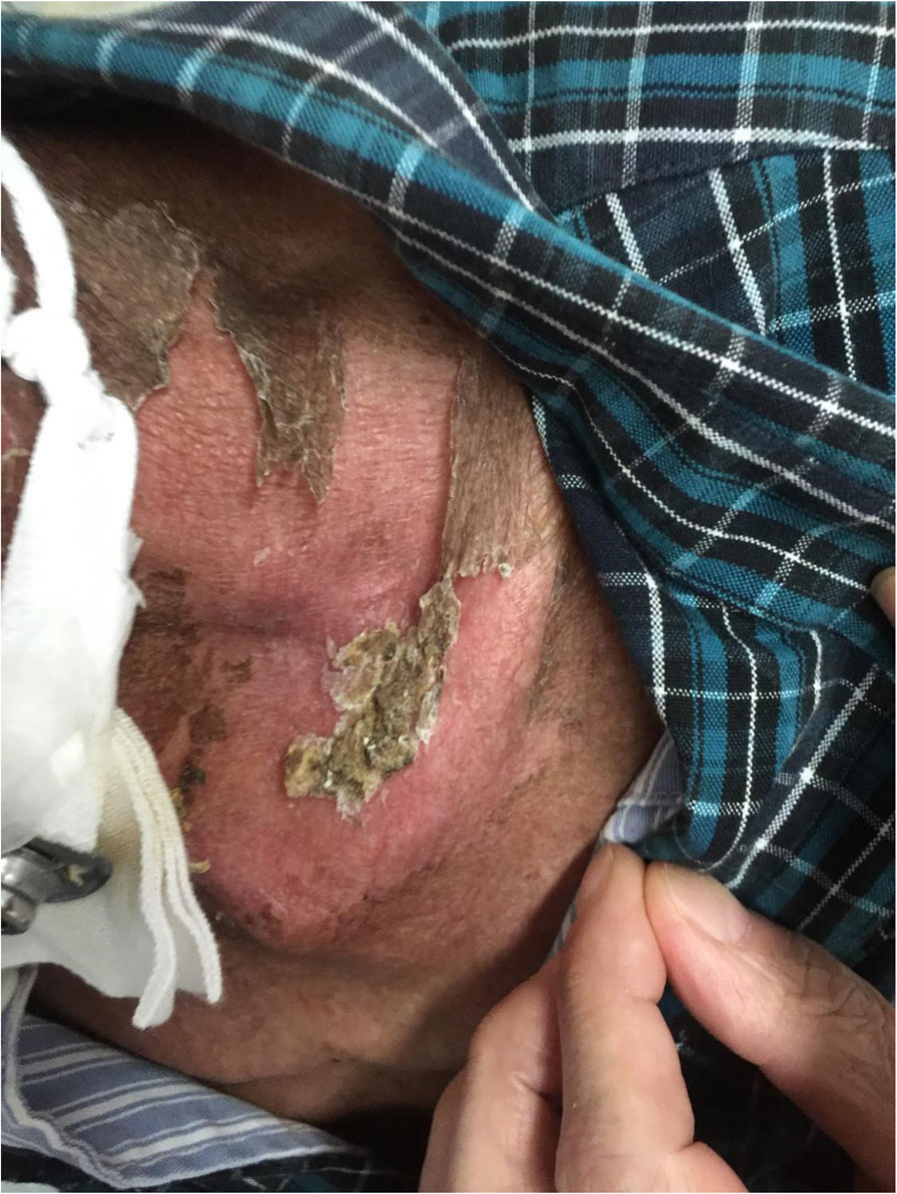
Patient with severe radioactive dermatitis, the patient stated that skin was painful, and skin surface ulceration and secretion could be seen. The seventh irradiation

**Fig. 6:**
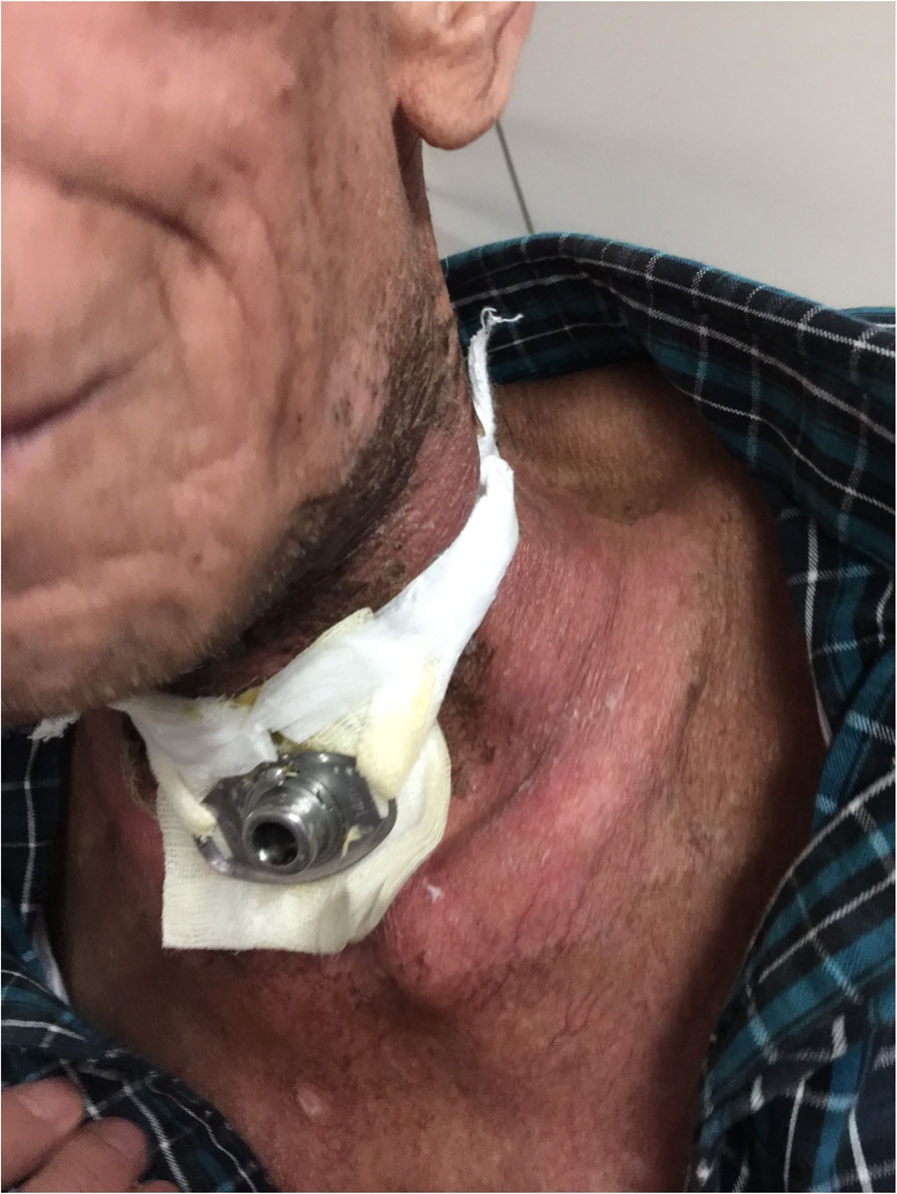
Patient with severe radioactive dermatitis, the patient stated that skin was painful, and skin surface ulceration and secretion could be seen. The eighth irradiation, and the new skin was basically formed

**Fig. 7:**
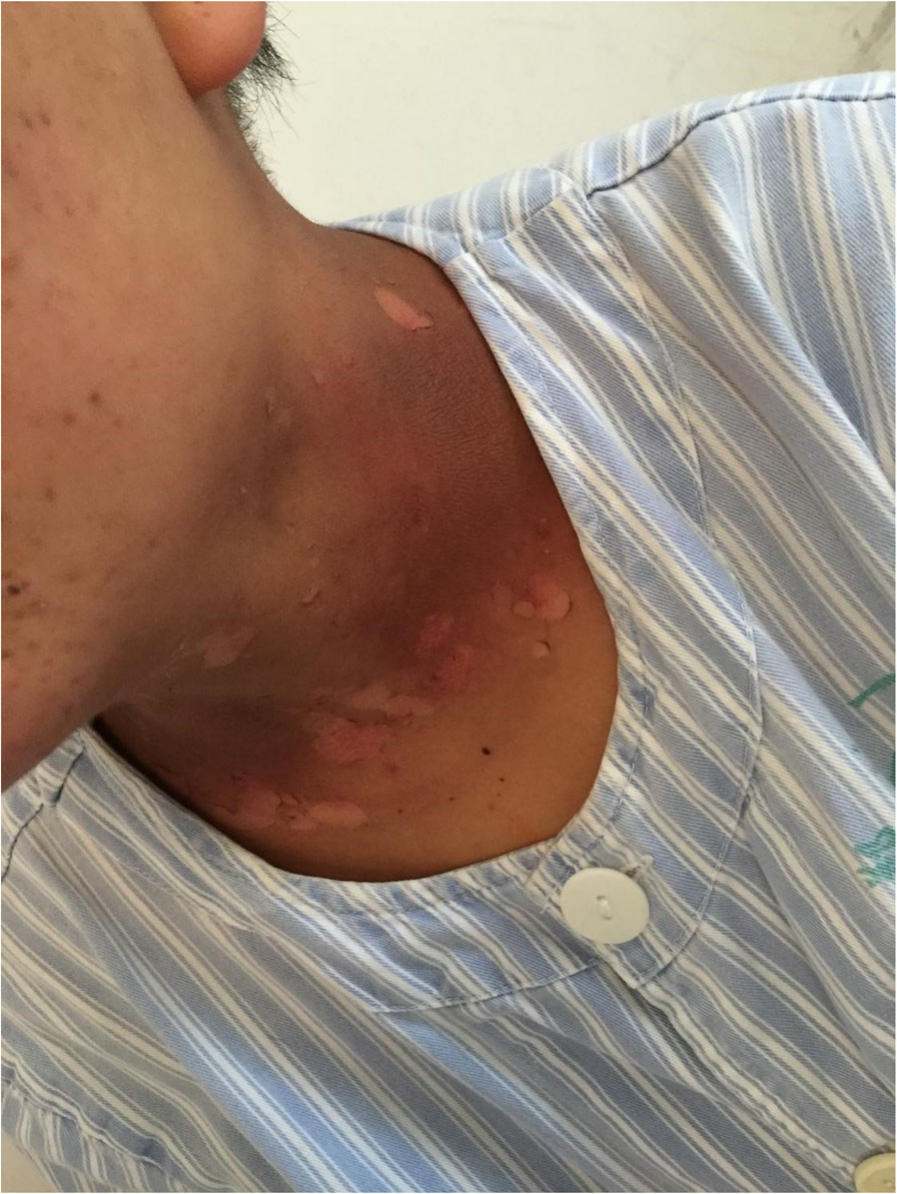
Patient with severe radioactive dermatitis, the patient stated tingling in the irradiation area, the skin was gray, with multiple small ulcerations and a small amount of seepage

**Fig. 8:**
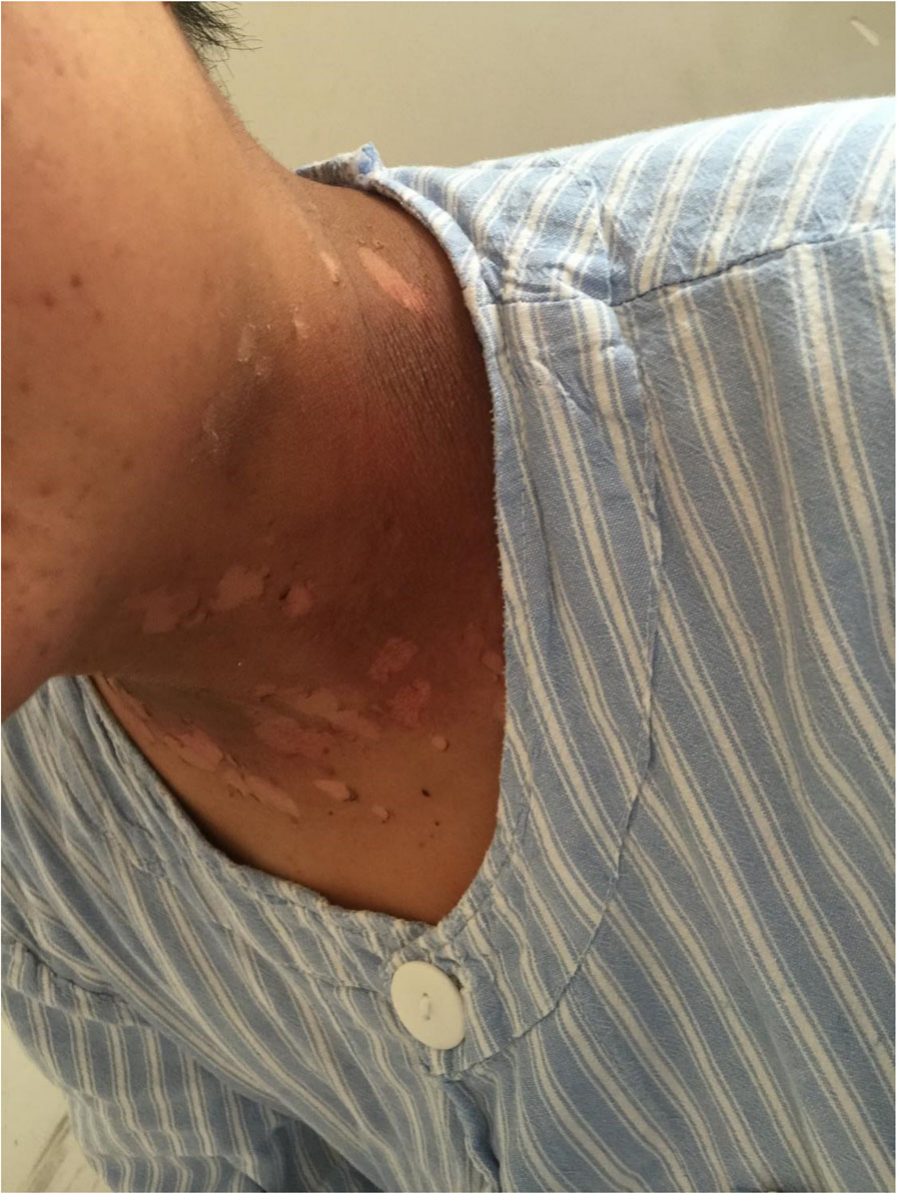
The second irradiation

**Fig. 9:**
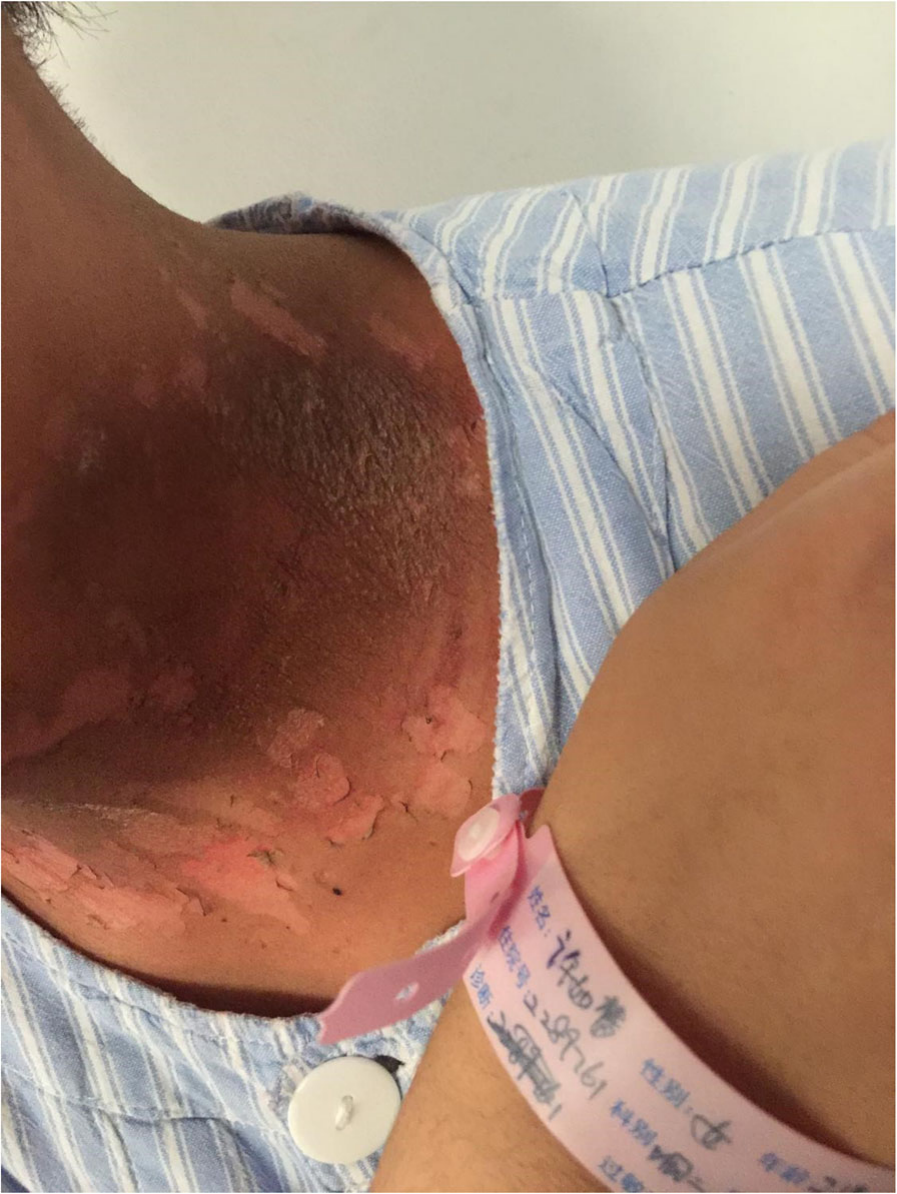
The forth irradiation, the skin was dry and part of started to peel off

**Fig. 10:**
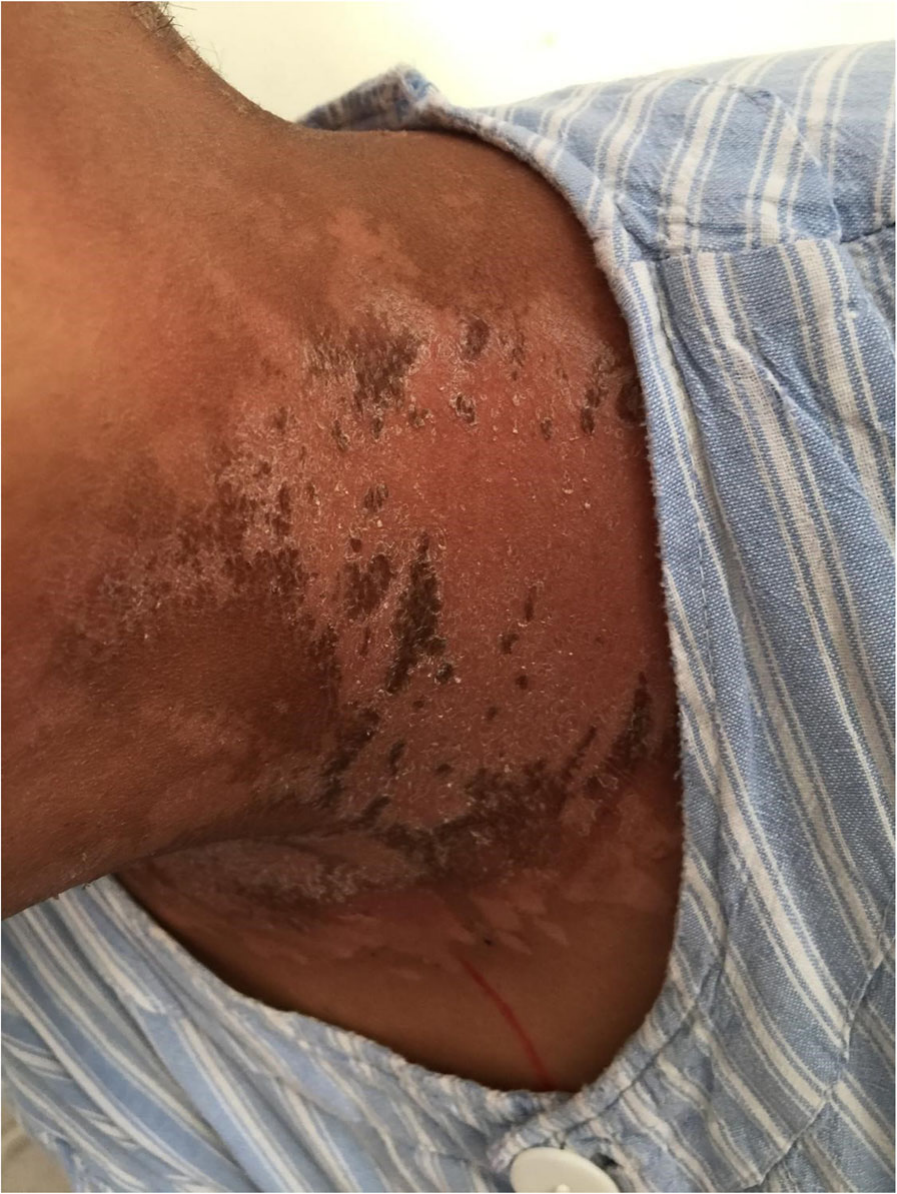
The fifth irradiation

**Fig. 11:**
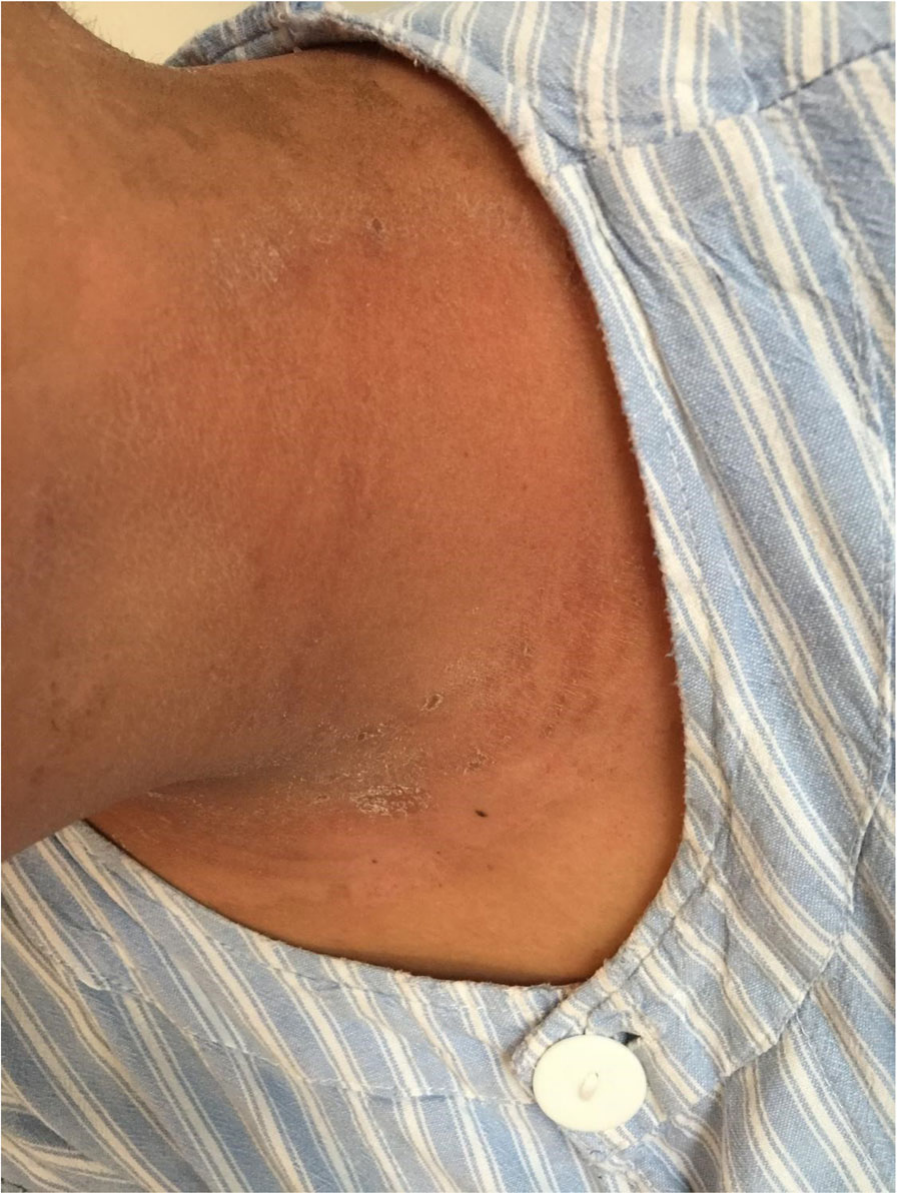
The seventh irradiation, and the new skin was formed

**Fig. 12:**
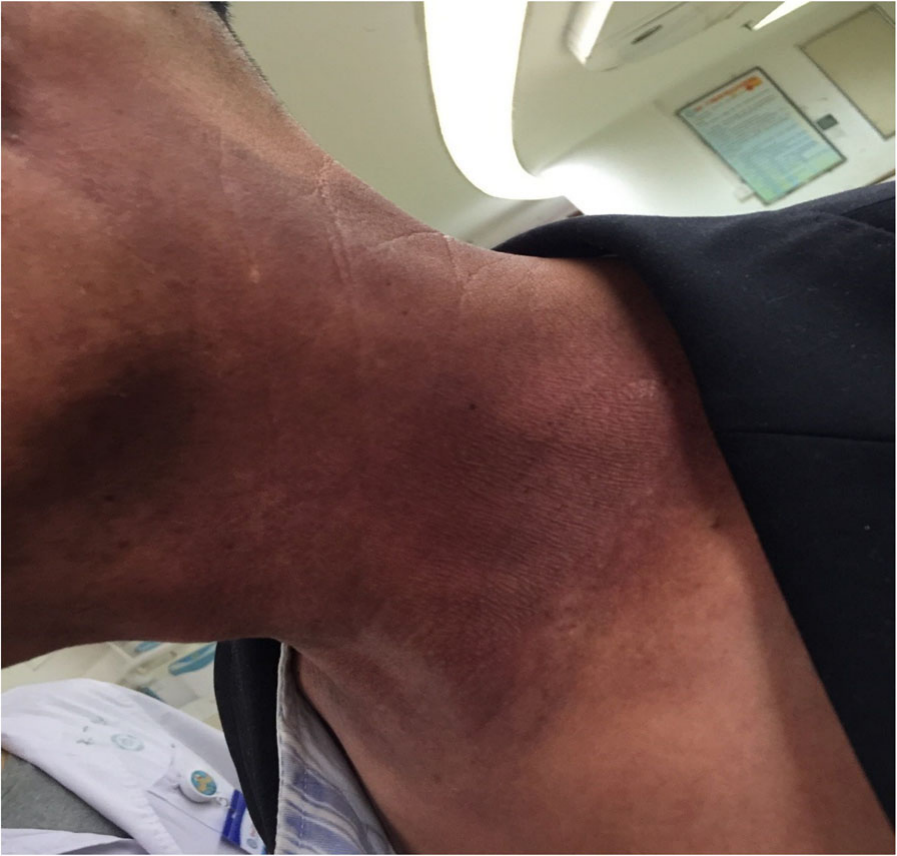
Patient with mild radioactive dermatitis, the patient stated mild tingling in irradiation area, the skin was black, without ulceration

**Fig. 13:**
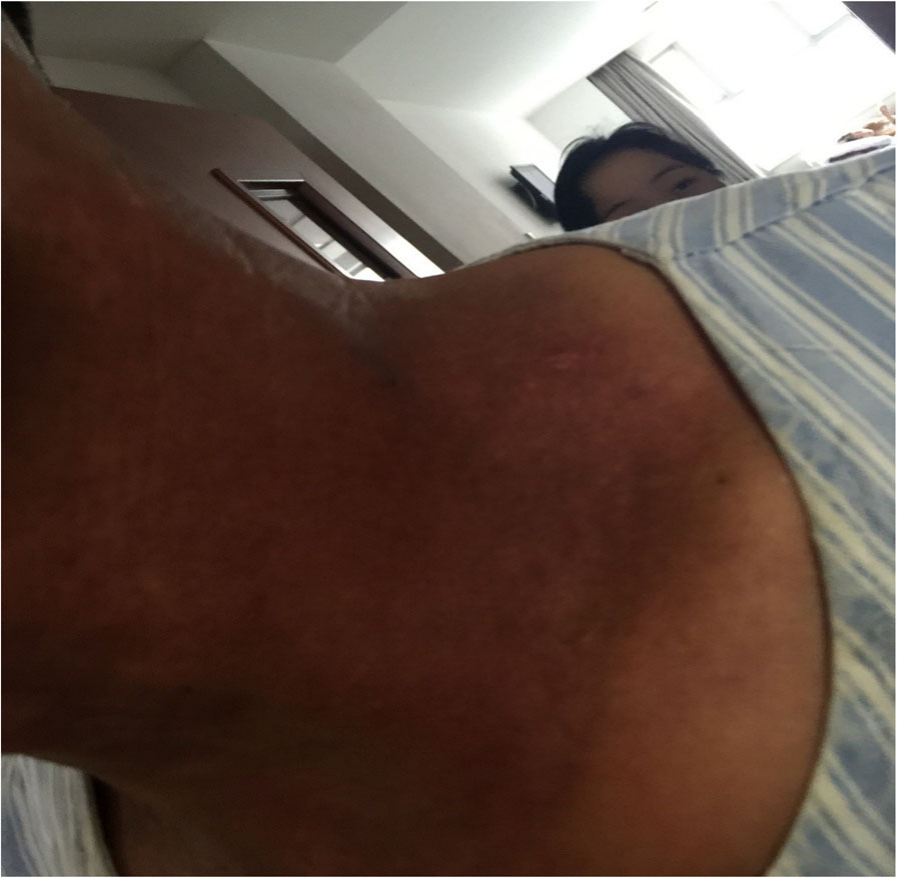
The first irradiation

**Fig. 14:**
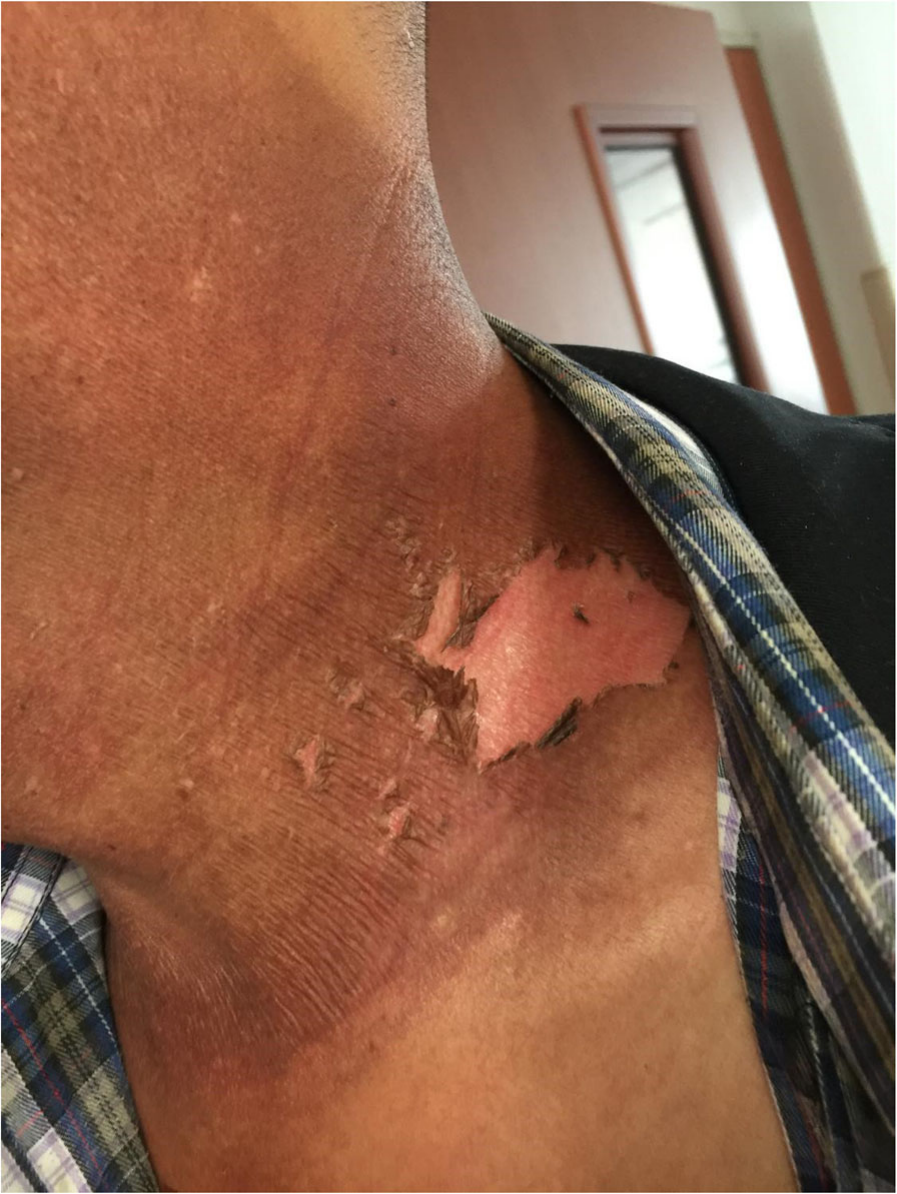
The fourth irradiation, the black skin was scabbed and peeled off

**Fig. 15:**
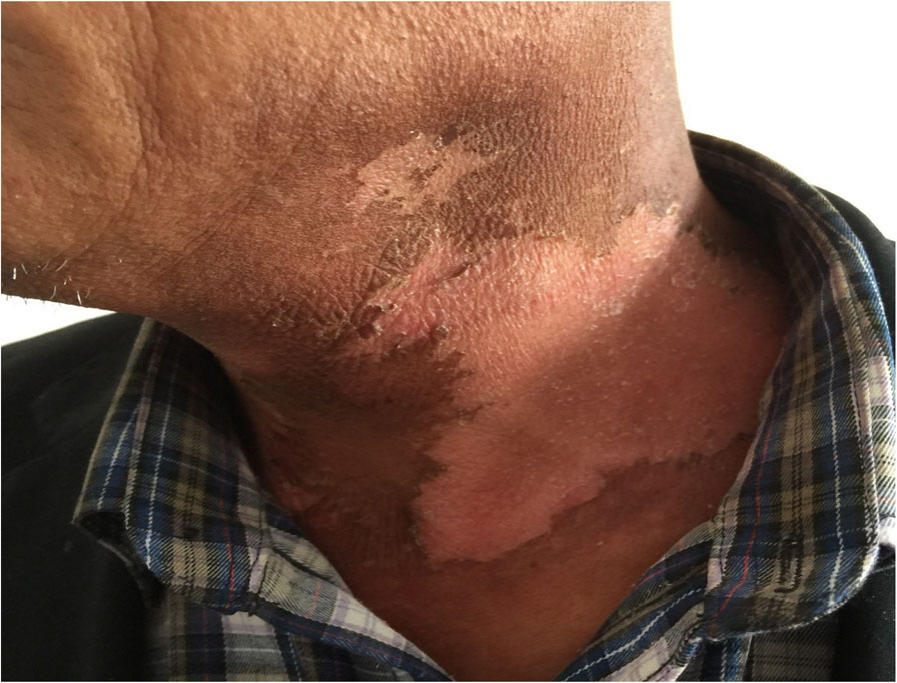
The sixth irradiation, and the new skin was formed

The results of this study indicated that close-range RLPT had a good therapeutic effect on RD. After the application of RLPT, the degree of RD reaction in the experimental group was lighter than that in the control group (*P* < 0.05, Table 2). This indicated that RLPT promoted the healing of inflammation and shortened the healing time, which could not only reduce the complications such as infection caused by mucosal damage, but also further improve the control rate of tumor. The results of pain score in the patients who participated in the experiment showed (Table 3) that the wound pain score of the experimental group was significantly lower than that of the control group, with a significant difference (*P* < 0.05). It is suggested that RLPT can effectively relieve or alleviate wound pain and reduce the pain caused by skin reaction (Figs. 5, 6, 7, 8, 9, 10, 11, 12, 13, 14, and 15).

## Conclusions

In conclusion, RLPT can accelerate the healing ability of wound and significantly shorten the healing time. It can not only relieve the pain of patients’ wounds and promote the healing of inflammation and ulcer, but also guarantee the smooth progress of the patients’ radiotherapy and improve their quality of lives, which is worth the popularization and application in the clinical practice.

## Abbreviations

HNC: Head and neck cancer
NPC: Nasopharyngeal carcinoma
NRS: Numerical rating scale
RD: Radioactive dermatitis
RLPT: Red light phototherapy
